# Survival and quality of life in patients acquiring acute kidney injury in the first 24 hours of ICU admission

**DOI:** 10.1186/cc13245

**Published:** 2014-03-17

**Authors:** I Soliman, L Peelen, D De Lange, D Van Dijk

**Affiliations:** 1 University Medical Center, Utrecht, the Netherlands

## Introduction

Survival and quality of life (QoL) in ICU patients with acute kidney injury (AKI) have been repeatedly reported as poor [1-3]. It is unknown whether early AKI, occurring during the first 24 hours of ICU treatment, is also a strong predictor of poor long-term outcome. Our aim was to describe the long-term outcomes of this specific group of ICU patients.

## Methods

All patients admitted to our mixed ICU from July 2009 to May 2012 with early AKI were included. All survivors received the EuroQoL EQ-6D-3L questionnaire. Early AKI was defined as creatinine >1.5 mg/ dl and urine output <150 ml/8 hours. Poor ICU outcome was defined as either death or EuroQoL index <0.4 at 1-year follow-up.

## Results

**Figure 1:**
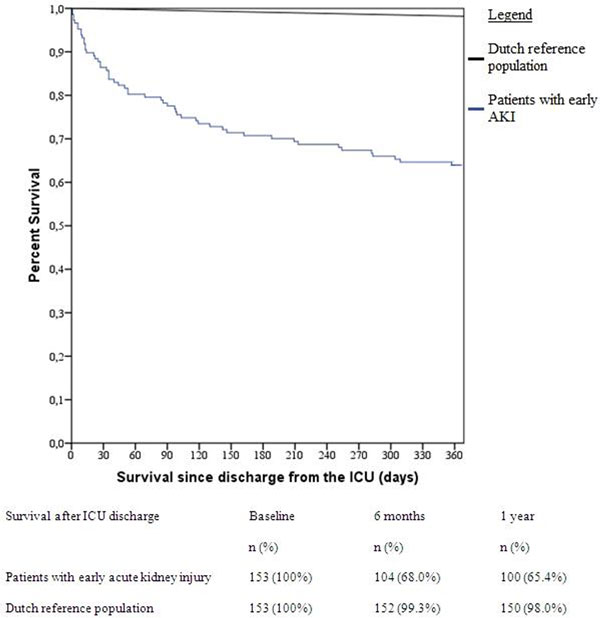
**Survival**.

Out of 5,934 admissions, 269 patients were identified with early AKI. After ICU discharge a large and significant difference in survival between included patients and the Dutch population, matched for age and gender, was seen (*P *< 0.001). The median QoL index in surviving patients was 0.65 (interquartile range (IQR) 0.45 to 1.00), versus 0.86 (IQR 0.84 to 0.89) in the Dutch population (*P *< 0.001). Low QoL was found in 11/59 (18.6%) survivors. In total, poor ICU outcome was seen in 171/269 (63.6%) patients. See Figure 1.

## Conclusion

Patients developing AKI in the first 24 hours of ICU stay are prone to poor outcome. Future research into prognostic factors for ICU patients should include early AKI in their models.
